# Full Genome Sequence of the Western Reserve Strain of Vaccinia Virus Determined by Third-Generation Sequencing

**DOI:** 10.1128/genomeA.01570-17

**Published:** 2018-03-15

**Authors:** István Prazsák, Dóra Tombácz, Attila Szűcs, Béla Dénes, Michael Snyder, Zsolt Boldogkői

**Affiliations:** a Department of Medical Biology, Faculty of Medicine, University of Szeged, Szeged, Hungary; b Veterinary Diagnostic Directorate of the National Food Chain Safety Office, Budapest, Hungary; c Department of Genetics, School of Medicine, Stanford University, Stanford, California, USA

## Abstract

The vaccinia virus is a large, complex virus belonging to the Poxviridae family. Here, we report the complete, annotated genome sequence of the neurovirulent Western Reserve laboratory strain of this virus, which was sequenced on the Pacific Biosciences RS II and Oxford Nanopore MinION platforms.

## GENOME ANNOUNCEMENT

Poxviruses are generally brick-shaped (1), enveloped viruses that have a complex internal structure, including a relatively large double-stranded DNA genome and associated enzymes (2). In contrast to many other DNA viruses, poxviruses replicate and express their genomes within the cytoplasm rather than in the nucleus of the infected cell (3).

The vaccinia virus (VV) is a historically interesting and significant virus; it has been successfully used as a vaccine for immunization against human smallpox (4–6), which was declared eradicated in 1980 by the WHO thanks to global vaccination efforts (7). The linear genome of VV is approximately 190 kb in length; it is flanked by inverted terminal repeat sequences and encodes around 220 protein-coding genes (8). The most virulent strain of VV, the Western Reserve (WR) (9), was used for our study. The currently available third-generation (long-read) sequencing platforms, namely, the Pacific Biosciences (PacBio) RS II system and the Oxford Nanopore Technologies (ONT) MinION system, were used for the cDNA sequencing.

The CV-1 cell line (ATCC CCL-70) was infected with the laboratory WR strain of VV. The poly(A) fraction of the purified RNA was converted to cDNA following the Isoform Sequencing (Iso-Seq) protocol for PacBio sequencing and the cDNA-seq protocol (1D strand switching cDNA by ligation) for the MinION process. Seventeen single-molecule real-time (SMRT) cells with P6-C4 chemistry, as well as two MinION flow cells, were run on the PacBio and ONT systems, respectively. The PacBio sequencing resulted in 59,154 reads, while 16,175 reads were obtained from the MinION runs. Our data revealed extreme transcriptional activity across the entire VV genome. Both of the sequencing platforms that we used in this study cover the full-length viral DNA. The average coverages were 289.3× (PacBio) and 43.6× (ONT), and the mean sizes of the aligned read lengths were 953 bp (PacBio) and 525 bp (ONT).

The PacBio raw reads were processed and mapped to the respective reference genome (GenBank accession no. NC_006998) with the BLASR long-read mapper (https://github.com/PacificBiosciences/blasr). GMAP (10) was also used for aligning the reads. The MinION sequencing reads were mapped to the reference genome by using Albacore version 1.2.6 software. The genome was assembled and the open reading frames were predicted by using Geneious software (11).

The complete genome sequence of the WR strain of VV is composed of 194,888 bp. The average G+C content of the genome is 33.3%. The virus contains 218 protein-coding genes. Our data show that the sequenced WR strain differs in 163 point mutations—among which were 58 nonsilent mutations—from the GenBank reference sequence. Eighteen insertions and four deletions were also detected.

The most common point mutation is a substitution of cytosine for thymidine. Most of the amino acid substitutions occurred in the C9L gene. A new coding sequence (A54L) was annotated. The majority of nucleic acid changes (21 events) were clustered in the F region of the viral genome. Mutations mainly occurred in viral genes playing a role in host defense modulation, transcription regulation, and viral replication.

### Accession number(s).

The complete and annotated genome sequence of the WR strain of VV has been deposited in the European Nucleotide Archive under accession no. LT966077.
